# Supplementary material for: Do Different Patterns of Employment Quality Contribute to Gender Health Inequities in the U.S.? A Cross-Sectional Mediation Analysis
Source: Int J Environ Res Public Health. 2022 Sep 7;19(18):11237. doi: 10.3390/ijerph191811237 (PMC9517248; doi:10.3390/ijerph191811237)
Supplement: Supplementary file 1 [file ijerph-19-11237-s001.zip › ijerph-1817873-supplementary.pdf]

Table S1. Conceptual dimensions and indicators used to operationalize employment quality (EQ) within the U.S. General Social Survey (GSS).

EQ Dimensions	Indicators	GSS Item(s)	Operationalization
**Employment stability** - whether the expectation of employment is ongoing, as opposed to time-fixed or project-dependent	Employment contract	Type of work arrangement	1) regular, permanent employment<br>2) independent contractor; working under a contractor; employment on an on-call basis, or paid by temporary agency
**Material Rewards** - labor income and employer-provided fringe benefits	Income level	Labor earnings	1) lowest quartile<br>2) second or third quartile<br>3) highest quartile
**Workers' rights & social protection** - labor protections provided to workers and the extent to which they are upheld in the workplace	Days of mandatory extra work hours	Combines two items asking a) days per month work hours beyond usual schedule and b) if these hours were mandatory	1) zero days<br>2) between 1 and 10 days<br>3) >11 days
**Working time arrangements** - timing, regularity, and quantity of working hours	Long working hours	Hours worked last week	1) <24 hours<br>2) 25-36 hours<br>3) 37-48 hours<br>4) >48 hours
	Working times regularity	Work schedule	1) day shift<br>2) afternoon or night shift<br>3) split, irregular, on-call, or rotating shifts
**Employability opportunities** - whether a worker is able to develop skills/abilities	Training opportunities	Opportunity to develop abilities	1) yes (‘very true’ or ‘somewhat true’)<br>2) no (‘not too true’ or ‘not at all true’)
**Collective organization** - whether employees have labor representation and open lines of communication with management	Have adequate training, equipment, information	Combines two items asking if workers a) have enough help and equipment, and b) enough information to get job done	1) yes (‘often’ or ‘sometimes’ responses for both of these items)<br>2) no (responses of ‘rarely’ or ‘never’ on at least one)
	Union representation	Belong to labor union	1) yes<br>2) no
**Interpersonal power relations** - relations between workers, co-workers, superiors, and employers	Employee involvement	Take part in work-related decisions that affect you	1) ‘often’<br>2) ‘sometimes’<br>3) ‘rarely’ or ‘never’
	Control over schedule	Combines two items asking a) ability of workers to change starting/finishing times and b) ability to take off work for personal/family reasons	1) high control (high or medium control on both items)<br>2) medium control<br>3) low control (low control on both items)
	Subjected to harassment/abuse	Combines two items indicating having been a) sexually harassed or b) threatened or harassed in any other way by anyone while at their job	1) yes ('yes' to either item)<br>2) no

Notes: Conceptual framework of employment quality and choice of indicators adapted from Van Aerden et al. (2014, 2016).

Table S2. Employment quality indicators by gender: Frequency (percent).

EQ indicator	Response category	Total N = 6367	Women n = 3405	Men n = 2962	p-value[a]
**Employment arrangement**	Regular/permanent	5606 (88)	3052 (90)	2554 (86)	<0.001
	Non-permanent	714 (11)	322 (9)	392 (13)	
	Missing	47 (1)	31 (1)	16 (1)	
**Income**	Lowest income quartile	1456 (23)	978 (29)	478 (16)	<0.001
	2nd/3rd income quartile	2928 (46)	1558 (46)	1370 (46)	
	Highest income quartile	1087 (17)	389 (11)	698 (24)	
	Missing	896 (14)	480 (14)	416 (14)	
**Days of mandatory extra work hours**	None	4915 (77)	2757 (81)	2158 (73)	<0.001
	1-10 days	897 (14)	424 (12)	473 (16)	
	11+ days	387 (6)	135 (4)	252 (9)	
	Missing	168 (3)	89 (3)	79 (3)	
**Working hours**	<24	646 (10)	429 (13)	217 (7)	<0.001
	25-36	834 (13)	599 (18)	235 (8)	
	37-48	3217 (51)	1738 (51)	1479 (50)	
	>48	1643 (26)	623 (18)	1020 (34)	
	Missing	27 (0)	16 (0)	11 (0)	
**Working times regularity**	Day shift	4658 (73)	2529 (74)	2129 (72)	0.017
	Afternoon/night shift	771 (12)	402 (12)	369 (12)	
	Split/irregular/rotating	895 (14)	445 (13)	450 (15)	
	Missing	43 (1)	29 (1)	14 (0)	
**Opportunity to develop abilities**	Very true	2217 (35)	1169 (34)	1048 (35)	0.003
	Somewhat true	2757 (43)	1431 (42)	1326 (45)	
	Not true	1336 (21)	771 (23)	565 (19)	
	Missing	57 (1)	34 (1)	23 (1)	
**Have adequate training, info, equipment**	Often/sometimes	5485 (86)	2915 (86)	2570 (87)	0.210
	Rarely/never	840 (13)	463 (14)	377 (13)	
	Missing	42 (1)	27 (1)	15 (1)	
**Union representation**	Union member	620 (10)	294 (9)	326 (11)	0.005
	Not union member	3627 (57)	1975 (58)	1652 (56)	
	Missing	2120 (33)	1136 (33)	984 (33)	
**Control over schedule**	High control	1615 (25)	822 (24)	793 (27)	0.017
	Medium control	1916 (30)	1018 (30)	898 (30)	
	Low control	1532 (24)	866 (25)	666 (22)	
	Missing	1304 (20)	699 (21)	605 (20)	
**Employee involvement**	Often involved	2483 (39)	1284 (38)	1199 (40)	0.071
	Sometimes involved	2434 (38)	1314 (39)	1120 (38)	
	Rarely/never involved	1407 (22)	780 (23)	627 (21)	
	Missing	43 (1)	27 (1)	16 (1)	
**Workplace harassment/threats**	Yes	753 (12)	463 (14)	290 (10)	<0.001
	No	5569 (87)	2914 (86)	2655 (90)	
	Missing	45 (1)	28 (1)	17 (1)	

Notes: [a] Chi square test comparing women and men.
